# COVID-19 contact tracing in Belgium: main indicators and performance, January – September 2021

**DOI:** 10.1186/s13690-022-00875-6

**Published:** 2022-04-13

**Authors:** Kristiaan Proesmans, Sharon Hancart, Toon Braeye, Sofieke Klamer, Emmanuel Robesyn, Achille Djiena, Frances De Leeuw, Romain Mahieu, Alex Dreuw, Naima Hammami, Dirk Wildemeersch, Laura Cornelissen, Dieter Van Cauteren

**Affiliations:** 1 Department of Epidemiology and public health, Sciensano, Brussels, Belgium; 2 European Centre for Disease Prevention and Control (ECDC), Stockholm, Sweden; 3 Agence pour une Vie de Qualité, Charleroi, Belgium; 4 Department of Infectious Disease Prevention and Control, Common Community Commission, Brussels-Capital Region, Brussels, Belgium; 5 Ministry of the German-speaking Community, Eupen, Belgium; 6 Agency for Care and Health, Infection Prevention and Control, Flemish Community, Brussels, Belgium

**Keywords:** COVID-19, Belgium, Contact tracing

## Abstract

**Background:**

Contact tracing is one of the main public health tools in the control of coronavirus disease 2019 (COVID-19). A centralized contact tracing system was developed in Belgium in 2020. We aim to evaluate the performance and describe the results, between January 01, 2021, and September 30, 2021. The characteristics of COVID-19 cases and the impact of COVID-19 vaccination on testing and tracing are also described.

**Methods:**

We combined laboratory diagnostic test data (molecular and antigen test), vaccination data, and contact tracing data. A descriptive analysis was done to evaluate the performance of contact tracing and describe insights into the epidemiology of COVID-19 by contact tracing.

**Results:**

Between January and September 2021, 555.181 COVID-19 cases were reported to the central contact center and 91% were contacted. The average delay between symptom onset and contact tracing initiation was around 5 days, of which 4 days corresponded to pre-testing delay. High-Risk Contacts (HRC) were reported by 49% of the contacted index cases. The mean number of reported HRC was 2.7. In total, 666.869 HRC were reported of which 91% were successfully contacted and 89% of these were tested at least once following the interview. The estimated average secondary attack rate (SAR) among the contacts of the COVID-19 cases who reported at least one contact, was 27% and was significantly higher among household HRC. The proportion of COVID-19 cases who were previously identified as HRC within the central system was 24%.

**Conclusions:**

The contact-tracing system contacted more than 90% of the reported COVID-19 cases and their HRC. This proportion remained stable between January 1 2021 and September 30 2021 despite an increase in cases in March–April 2021. We report high SAR, indicating that through contact tracing a large number of infections were prospectively detected.

The system can be further improved by (1) reducing the delay between onset of illness and medical consultation (2) having more exhaustive reporting of HRC by the COVID-19 case.

**Supplementary Information:**

The online version contains supplementary material available at 10.1186/s13690-022-00875-6.

## Background

In Belgium, as in the rest of the world, the severe acute respiratory syndrome coronavirus 2 (SARS-CoV-2) causing coronavirus disease 2019 (COVID-19) resulted in important challenges to monitor and contain the spread of this new virus. From March 2020 onwards, the number of confirmed COVID-19 cases quickly increased, with widespread community transmission throughout the country and important numbers of COVID-19-associated hospitalizations and mortality [1–3]. As a result, non-pharmaceutical interventions (NPI) were implemented, among which a lockdown from March 14, 2020 [4]. A fast and effective contact tracing system, able to process large volumes of cases, together with an increase in the testing capacity, were identified as key public health tools to limit transmission, especially after lifting the lockdown. A performant system of testing and tracing was necessary to swiftly identify newly infected COVID-19 cases and their close contacts: people they had contact with during their infectious period. Contact tracing also included the quarantine and testing of high-risk contacts (HRC), while the contact center also had a role in informing the COVID-19 cases about their isolation. This combination of measures can lead to the interruption of transmission chains [5, 6]. The initial phase of the epidemic had highlighted the capacity limitations of traditional contact tracing performed by the regional public health services. Therefore, the regional authorities (Brussels-Capital, Flanders, Wallonia) together with the German-speaking community within Wallonia, decided to organize one centralized technical contact tracing platform that could be used by different regional call centers. This new system aimed to be operational by May 11, 2020, when lockdown measures were lifted [7]. An exception was made for companies and collectivities such as hospitals, schools, and long-term care facilities. In these settings, only HRC outside these collectivities (‘private contacts’) were followed by the central tracing, while local contact tracing of HRC within the collectivities was carried out by medical services, and thus not covered by this central tracing system. Moreover, in September 2020 the Belgian corona-app (Coronalert) was launched. This application allows COVID-19 cases to report their positive test and to warn their contacts anonymously. Via Bluetooth, the smartphone will exchange encrypted random identifiers with other devices. These identifiers, provide information solely about duration and distance of an encounter and allow a user to inform anonymously its contacts that used the app in case of a COVID-19 diagnosis [8–11].

In this paper, we describe the performance of the COVID-19 contact tracing system in the first 9 months of 2021 in Belgium, using indicators that are internationally recommended [12, 13]. Our objectives were to identify (i) the completeness and timeliness with which COVID-19 cases were captured by the system (ii) the proportion and timeliness with which their HRC were contacted. (iii) the proportion of HRC that were tested and those that became new COVID-19 cases (HRC who tested positive) and (iv) the proportion among all new COVID-19 cases which were registered as HRC in the central system.

Furthermore, we describe the characteristics of COVID-19 cases (age group, sex, symptoms, suspected place of infection, the use of the coronalert app) and the impact of the COVID-19 vaccination campaign on contact tracing from January to September 2021.

## Methodology

### Data sources

Three data sources were used: the laboratory test database, the contact tracing database, and the vaccination database. All three databases were linked at the person level via a unique pseudonymized national registry number (NRN) [14]. This NRN is the unique identification number with which a person is identified in, among other things, the health and social sector and is available for all Belgian residents. For non-Belgian residents (e.g. tourists) a “BIS” number can be created by a physician when necessary.

The test database includes test prescriptions and results reported by laboratories (molecular or antigen tests), physicians (molecular or antigen tests), and pharmacies (antigen tests). All test results were reported to Sciensano, the national institute of health.

The contact tracing database includes results of interviews (calls) with COVID-19 cases about possible COVID-19 related symptoms (anosmia, cough, headache, runny nose, muscle pain, sore throat, fever, and diarrhea), self-reported suspected place of infection, and their contacts during the infectious period (defined as 2 days before until 10 days after onset of symptoms – in case of no symptoms at the time of sampling, the date of sampling was used) [15]. Part of the index cases were asked question about the use of the CoronApp. Data from the COVID-19 cases and HRC were centralized in a contact tracing data warehouse.

The Belgium COVID-19 vaccination registry (Vaccinnet+) [16] was used to monitor the vaccination status of index cases and high-risk contacts over time.

Testing and quarantine measures over time were extracted from official governmental communications (Supplementary Table 1).

### Case definitions

A COVID-19 “index case” was defined as a person with a positive molecular or antigen test. Contact tracing could also be requested by a physician for a patient without a test result or if he judges that it was a false negative result given his knowledge of the history and clinical background of the patient. In order to take into account only new infectious episodes and to avoid re-contacting the same person during one episode, persons with a previous infection in the past 56 days (until 01 April 2021) or 90 days (since 01 April 2021) were excluded (Supplementary Table 1). Index cases were reported to the contact center in order to initiate contact tracing [3]. Contacts were classified as HRC based on the type (e.g. direct physical contact, face-to-face contact at < 1,5 m with/without the use of face mask) and the duration of exposure (e.g. less or more than 15 min), while exact definitions varied over time and depending on the exact context [17, 18].

### Data analysis

The test, vaccination, and contact tracing data were merged based on the pseudonymized NRN. The secondary attack rates (SAR) were estimated as the proportion of HRC tested positive among all HRC tested at least once, using test data up to 14 days after the interview. Three different vaccination statuses were considered: unvaccinated, partially vaccinated, and fully vaccinated. A person was considered fully vaccinated if a test was taken at least 14 days after the second dose of the Moderna® or AstraZeneca® vaccine, 7 days after the second dose of Pfizer®, or 21 days after the first dose of the COVID-19 Janssen® vaccine. If only one dose of the Pfizer®, Moderna®, or AstraZeneca® vaccine had been administered, the person was considered partially vaccinated. To study the effect of vaccination on testing and contact tracing in Belgium, fully vaccinated and unvaccinated HRC were compared concerning their proportion tested and the positivity ratio.

The datasets used in this analysis contain data related to index cases tested between January 01, 2021, and September 30, 2021. Differences in categorical distributions were evaluated by a chi-square test. Confidence intervals were estimated by Wilson’s score method. All analyses were performed in R software version 4.0.5 [19].

## Results

### Identification of index cases

**Fig. 1:**
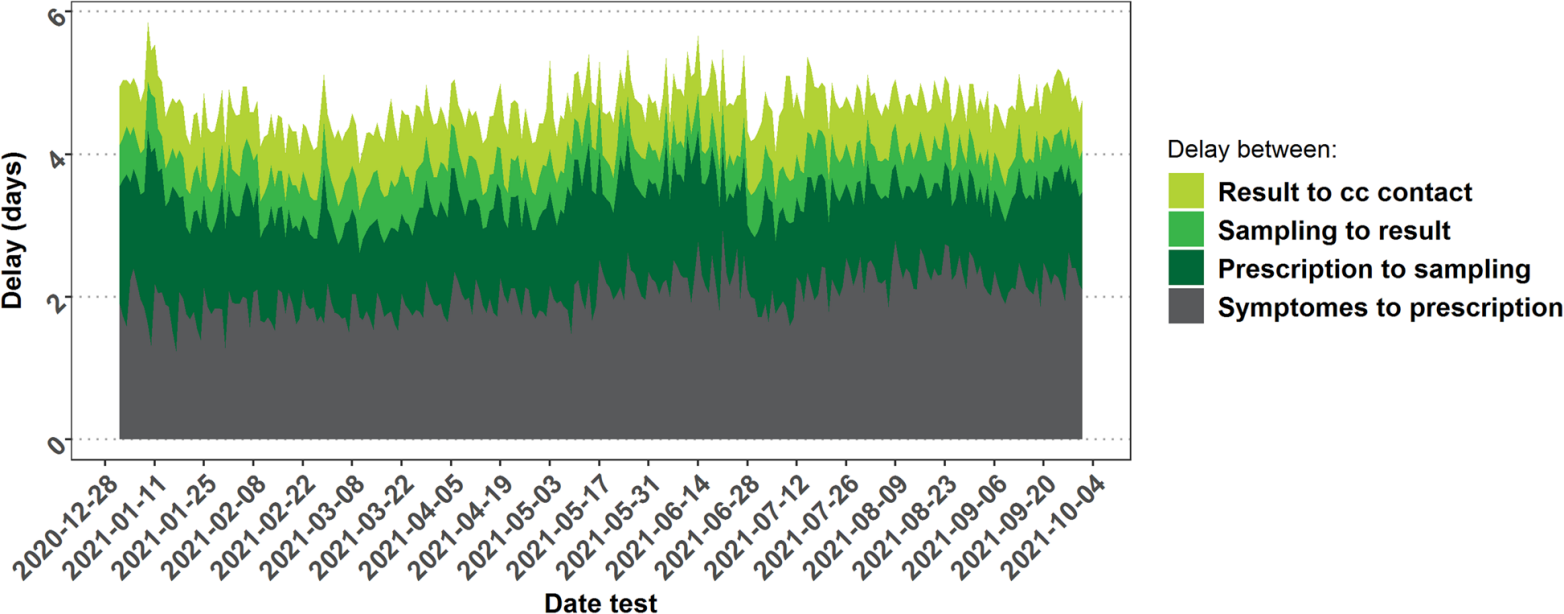
Mean duration of the onset of symptoms between the onset of symptoms and initiation of contact tracing by the contact center (CC), Belgium, January – September 2021 (*N* = 555.181)

Between January and September 2021, 555.181 index cases were reported to the contact center and 506.419 cases were contacted (Table 1) and 34.7% of the index cases reported a date of symptoms onset when a test was prescribed. The delay from symptom onset to interview is 4–5 days and can be divided into four periods. Figure 1 depicts the duration of each of these periods and their evolution over time. The first period, from symptom-onset to consultation with a healthcare provider, accounts for nearly half of the total delay for symptomatic persons. After a prescription was done by the healthcare provider, another 2–3 days were necessary for the complete process of sample collection, sample analysis, test result reporting, and contact tracing initiation by the contact center. Over the whole period, 40.7% of cases were contacted, by the contact center on the day of diagnosis. One day after the diagnosis, 79.9% of the cases were contacted. Overall 8.8% could not be reached by phone for an interview.

**Table 1:** Numbers and characteristics of index cases contacted by the contact center (CC), Belgium, January – September 2021 (Avg No. = average number, HRC = High-Risk Contact)

Month	Number	Symptomatic (%)	Fully vaccinated (%)	Reporting at least one HRC (%)	Avg No. HRCs among cases reporting HRC
January	51.834	75.8	0.0	46.5	2.6
February	51.086	71.3	0.7	47.4	2.7
March	102.144	70.9	0.8	46.4	2.7
April	90.667	70.2	1.5	47.7	2.6
May	57.741	71.0	2.6	49.5	2.8
June	15.970	70.8	4.9	52.1	2.8
July	33.353	76.4	12.4	54.8	2.8
August	52.045	75.6	24.9	48.9	2.7
September	51.579	73.5	34.6	53.8	3.0
**Total**	**506.419**	**72.4**	**7.8**	**48.8**	**2.7**

Table 1 illustrates the number of contacted cases and characteristics by month. Additional characteristics (distribution by age group, gender, region) can be found in Supplementary Table 2.

Most index cases (72.4%) reported possible COVID-19 symptoms. The most frequently reported symptoms, when contacted by the contact center, were headache (41.6%), cough (38.4%), runny nose (34.9%), muscle pain (32.4%), sore throat (28.8%), and fever (28.8%). Anosmia was reported by 17.8% of the cases, and 10.7% reported diarrhea.

Overall the app Coronalert was installed by 3.7 million persons in Belgium, which represents 32.1% of the population or 41% with people with a smartphone [20]. Among the contacted index cases 56.1% (*n* = 284.282) accepted to answer additional questions on the use of the app. Among these cases, 28.2% (*n* = 80.010) reported that they had installed the Coronalert app, of which 43.3% (*n* = 34.651) reported that they had used the app to alert contacts after the positive test. The proportion of installation and usage remained stable throughout the study period and was the highest among the surveyed working population, 20–65 years old: 30.6% of those installed the app, of which 44.4% used it to warn their contacts.

**Fig. 2:**
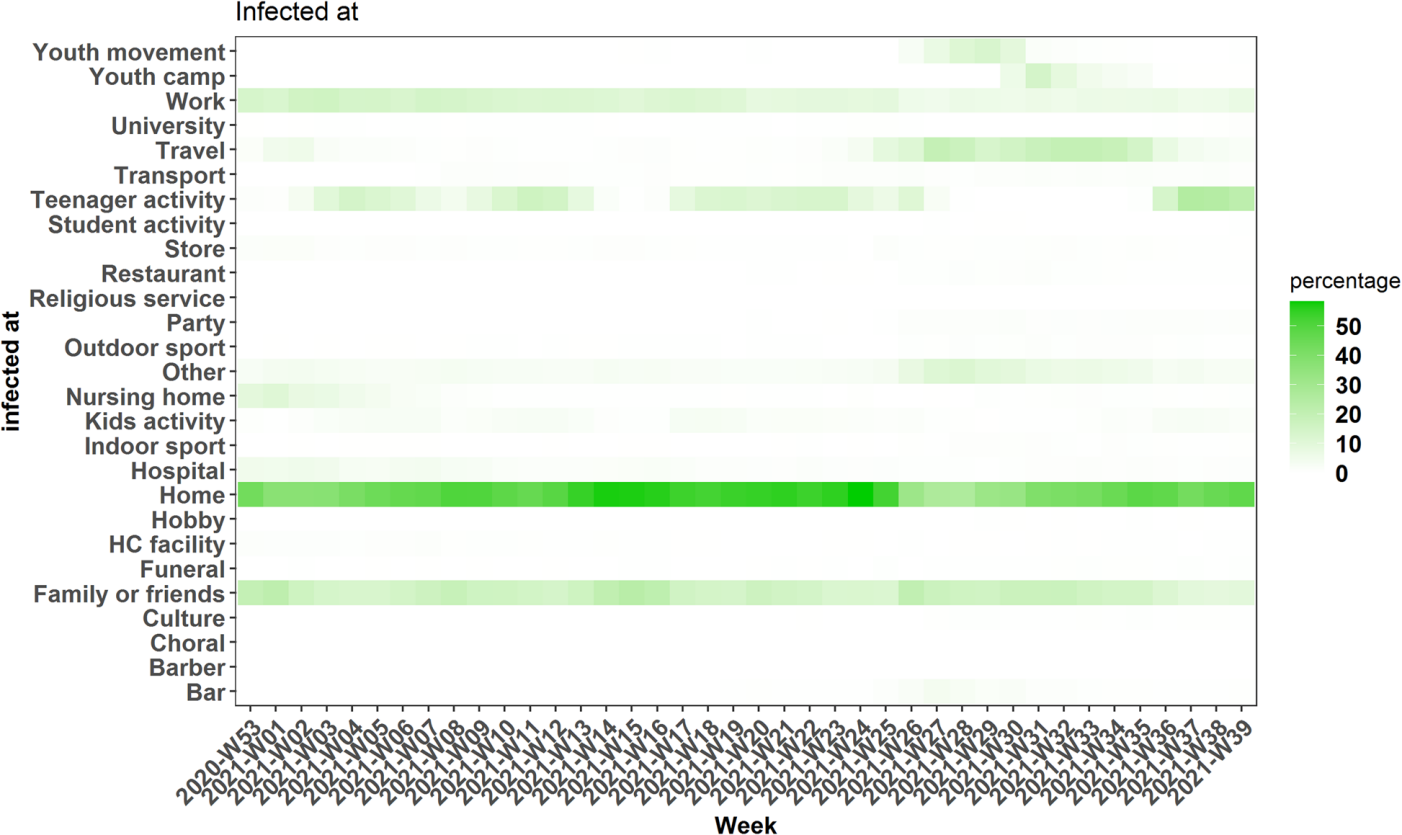
Self-reported places of infection among the index cases with strong suspicion, Belgium, January – September 2021. Color-scale depicts the distribution (%) over the categories within a week

Regarding the self-reported, most probable place of infection, 53.2% had a strong suspicion of the place where they could have been infected. Over the entire study period, the most common place of infection remained the household (47.5%). Travel and youth movements were more frequently reported during the summer holidays (week 26–35). Other settings that were reported were family or friends (15.9%), work (10.7%), and teenage activities (including schools) (9.1%). The evolution over time is given in Fig. 2.

**Fig. 3:**
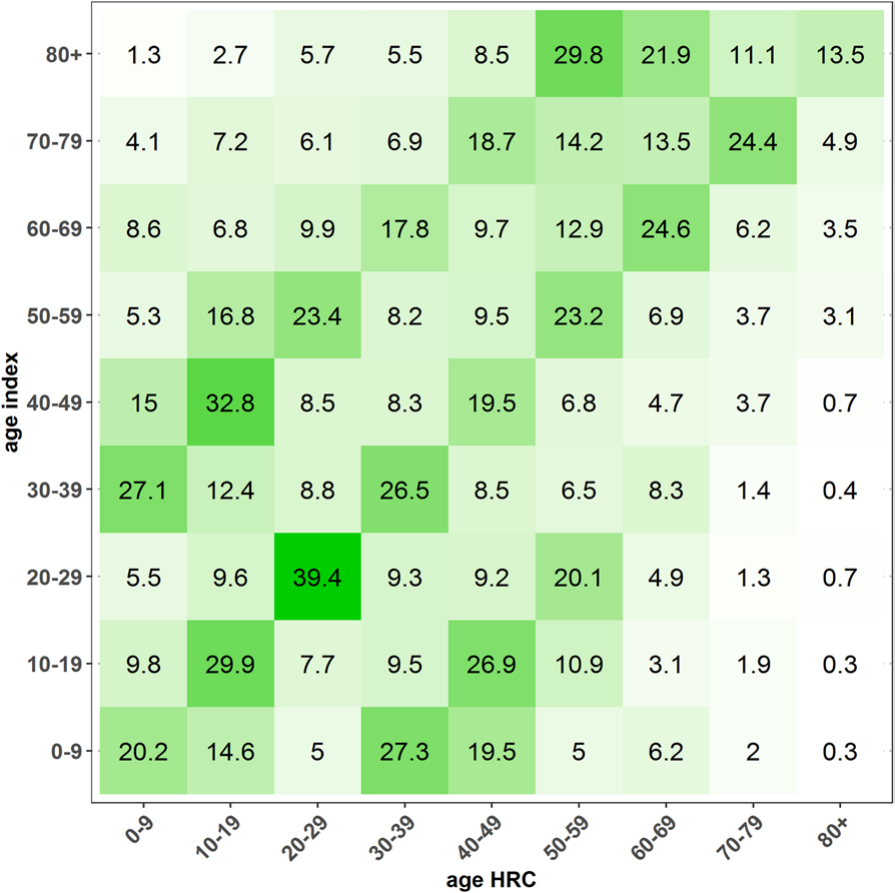
The age distribution of the high-risk contacts (HRC) for the different age groups of index cases, Belgium, January – September 2021 (numbers represent row-percentages) (*N* = 562.129)

The age of the HRC and index cases is presented in Fig. 3, showing the social dynamics. High-risk contacts occur most often between people of the same age group. A generation gap, visible among groups that differ 20–30 years, can be interpreted as contacts between parents and children or other contacts with a person of the generation of ones parent or ones child.

### Identification of high-risk contacts (HRC)

Among the contacted index cases, 247.351 (48.8%) reported at least one HRC, and among those on average 2.7 HRC were reported (Table 1). At least 1 non-household HRC was reported by 81.795 (16%) of the contacted index cases, and among those on average 2.2 non-household HRC were reported. Overall, 666.869 high-risk contacts (HRC) were reported of which 608.556 (91.3%) were successfully contacted. An NRN was available for 573.418 HRC (94%). Table 2 illustrates the evolution of these numbers by month and the characteristics of the contacted HRC. From the HRC, 76.0% were contacted the same day they were reported to the CC, 13.0% were contacted 1 day later and 8.7% were not reached.

**Table 2:** Numbers and characteristics of high-risk contacts (HRC), Belgium, January – September 2021

	All HRC			HRC with NRN			
Month	Number	Contacted HRC (%)	HRC with NRN (%)	Symptomatic at contact with CC (%)	Household contact (%)^**a**^	Fully vaccinated (%)	The secondary attack rate (SAR) (%)^**b**^
**January**	61.936	94.2	87.6	16.3	69.0	0.1	28.0
**February**	64.245	94.1	87.5	17.2	67.9	0.6	30.6
**March**	126.325	91.3	84.7	17.8	65.4	2.1	31.2
**April**	111.521	89.0	83.2	15.5	63.3	3.7	33.0
**May**	78.989	90.7	79.4	13.3	65.4	5.8	26.2
**June**	23.139	92.1	88.6	10.2	69.4	11.2	20.5
**July**	50.130	88.1	87.4	9.4	61.3	33.1	19.0
**August**	67.916	91.0	89.9	10.9	68.8	47.5	24.2
**September**	82.668	92.3	90.9	13.6	71.3	55.8	22.2
**Total**	**666.869**	**91.3**	**86.0**	**14.5**	**66.5**	**17.0**	**27.4**

^a^Among HRC with documented household status (*n* = 540.457)

^b^Among HRC for which at least one test result is available (*n* = 509.201)

Most HRC (66.5%) were household contacts and 14.5% reported having possible COVID-19 related symptoms at the moment of the interview. Overall 88.8% of the HRC with known NRN were tested at least once following the interview and the SAR among them was 27.4%. The SAR was significantly higher among household HRCs compared to non-household contacts (33.8% CI:33.7–34.0 vs 16.1% CI:15.9–16.3, *p* < 0.001) and among symptomatic HRCs compared to asymptomatic HRCs (49.5% CI:49.1–49.9 vs 23.9% CI:23.8–24.0, p < 0.001). Over the whole period, HRC, which were linked to an index case and registered as such in the tracing database, and for whom an NRN was available, represented 6.2% of all reported laboratory tests and 24.0% of all new cases.

### The impact of COVID-19 vaccination

**Fig. 4:**
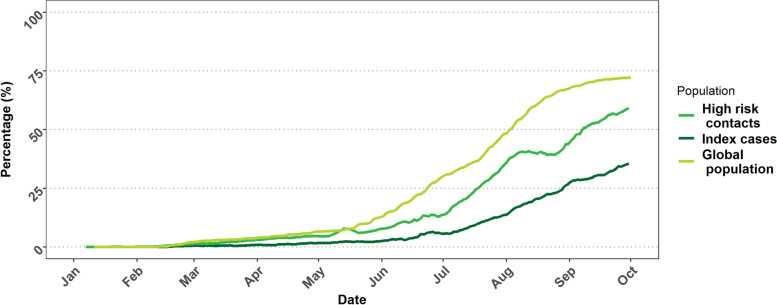
The 7-day averaged proportion of fully vaccinated among the index cases, high-risk contacts, and the Belgian population, Belgium, January – September 2021

The evolution over time of the proportion of people fully vaccinated among index cases, high-risk contacts, and the total Belgian population is shown in Fig. 4. At the end of September 2021, 73.1% of the population was fully vaccinated whereas this proportion was 35.5% among the index cases and 59.1% among HRC.

Between June 24, 2021, and August 31, 2021, the testing strategy was different depending on the HRC vaccination status (for fully vaccinated HRC, if the first test was negative, no second test was required). Since September 01, 2021, the testing strategy did no longer depend on the vaccination status: a first PCR test was required as soon as possible after identification as HRC, and a second test was required 7 days after the last exposure (if the first test was negative), or sooner if symptoms appeared (Supplementary Table 1).

The proportion of adult HRC that underwent a first or second test and the corresponding positivity ratio is summarized in Table 3, by vaccination status. The proportion of HRC that got tested at least once was higher among the fully vaccinated compared to unvaccinated (92% CI:91.7–92.3 vs 85.2% CI:84.5–85.8). In contrast, the positivity ratio was significantly higher among the unvaccinated compared to the fully vaccinated HRC for both the first (27.4% CI:26.5–28.3 vs 8.8% CI:8.5–9.1, *p* < 0.001) and second test (19.9% CI:18.8–21.1 vs 9.3% CI:8.9–9.7, *p* < 0.001). The in overall positivity ratio among unvaccinated HRC was also significant higher than among fully vaccinated HRC (37.4% CI:36.4–38.4 vs 14.2% CI:13.8–14.5, *p* < 0.001).

**Table 3:** Proportion of HRC tested once and twice among all HRC, and the corresponding positivity ratio by vaccination status, adult population (minimum 18 years), Belgium, September 1st and September 30th 2021 (T1 = first test, T2 = second test)

HRC		Total HRC (tested and non-tested)^**c**^	T1 tested	T1 positive	T2 tested	T2 positive	Total Positive HRC
Fully vaccinated	N	38.575	35.491	3.124	20.478	1.901	5.025
	%	*100%*	*92.0*	*8.8*	*63.2*	*9.3*	*14.2*
Unvaccinated	N	10.779	9.181	2.512	4.637	922	3.434
	%	*100%*	*85.2*	*27.4*	*69.5*	*19.9*	*37.4*
Total	N	49.354	44.672	5.636	25.115	2.823	8.459
	%	*100%*	*90.5*	*12.6*	*64.3*	*11.2*	*18.9*

^c^Including the HRC of which the index case was sampled between September 1st and September 30th 2021. 1.165 HRC were excluded because of their partial vaccinated status

## Discussion

To interrupt onward COVID-19 transmission it is essential to swiftly identify cases with prompt identification of their exposed (high risk) contacts and the application of appropriate measures by cases and contacts. As the generation interval (the delay between an index case becoming infected and passing on the infection to a secondary case) of COVID-19 has been reported to be on average around 4–5 days [21, 22] the whole process of contact tracing should be as quick as possible. Additionally, it has been shown that cases become infectious before symptom onset, further reducing the time window to act if cases are identified after symptom onset [23–26].

The average delay between symptom onset and contact tracing initiation was around 5 days. This delay remained stable over the study period despite an increase in cases in March–April 2021. The longest delay is the time between the onset of symptoms and the consultation of a healthcare professional. The duration between the onset of symptoms and the testing by a healthcare professional is 3–4 days. This is slightly higher than the 2.8 days delay reported by France [27] but shorter than the 10.2 days by Brazil [28]. However, data is not available for many countries and comparison is difficult due to differences in the organization of the health care system and the epidemical situation when these studies were carried out.

Modeling studies indicate 4–5 days between symptom onset of the index case and contact tracing initiation as an important tipping point for contact tracing effectiveness [29, 30]. Although efforts have been made to speed up the testing and tracing process to its maximum further improvements are essential, in particular, shortening the time between symptom onset and a positive test result. Frequent and clear communication towards the general population about the importance of rapid testing is needed to stimulate the public to get tested as soon as possible when COVID-19 symptoms occur and respect immediate isolation awaiting the test result. Also, the threshold for testing needs to be kept as low as possible. Early November 2021, an online self-assessment questionnaire was implemented in Belgium allowing persons with mild symptoms to be tested without consultation [31]. This new tool could help to improve the speed of identification of cases and may also help to decrease the burden on traditional healthcare services by creating an alternative test circuit. Such solutions may need further exploration and assessment, not at least with variants having an even shorter incubation period [32] and considering the upcoming phase of transition towards more sustainable long-term prevention, control, and surveillance strategies for SARS-CoV-2.

More than 9 out of 10 index cases were successfully contacted and most were contacted on the day of diagnosis. Similar results were obtained for HRC: more than 9 out of 10 were contacted and 3 out of 4 were contacted the same day as the index case. This shows the high coverage and operational efficiency of the contact tracing system. In addition, field agents could be deployed to visit at home index cases that could not be contacted by the contact center (not included in the results). Although the study period included the third wave, the results remained stable despite a strong dependency on human resources to conduct interviews. The system however remains susceptible to facing high volumes of cases at a short time, especially when rapid increases happen (“waves”). This can overload the system, and calling of all cases and HRC will become unfeasible. Less in-depth, more automated contact tracing techniques were prepared (e.g. online self-reporting of HRC by index cases, inform HRC by SMS). The performance of such automated systems remains to be evaluated, but the lack of a human aspect might negatively affect contact tracing effectiveness.

Among the contacted index cases 72% reported symptoms. The proportion of asymptomatic cases (28%) is rather high compared to the range reported in other studies (17–25%) [33].

Cough, headache, and a runny nose were the symptoms most often reported. Anosmia was reported by 18% of the cases, which is at the lower end of the frequency reported in the literature [34]. However, our study lacks a follow-up of asymptomatic cases (only information about symptoms experienced at the time of interview were included) which may result in an overestimation of the asymptomatic fraction and self-reported symptoms are prone to social desirability bias [35]. Interestingly, the symptomatic proportion remained stable over the entire study period, despite different predominant lineages over time and the roll-out of the vaccination campaign in the general population in 2021.

Among the index cases that answered the questions on the Coronalert app, 28% reported that they had installed the Coronalert app, and 43% of the users reported that they had used the app to alert contacts after the positive test. These results are in line with the general statistics reported in Belgium on the app use. Digital proximity tracing via apps is a novel and promising measure to reduce the spread of COVID-19, with the potential to complement regular contact tracing and enhance contact tracing effectiveness [30, 36]. However, a majority of citizens need to be willing to install and use such an app to be effective. The proposed uptake threshold of 60% of the population remained unattainable in most countries [37–39]. Another limitation of the system is that the context (e.g. face-mask-wearing) of the possible exposure could not be taken into consideration in the system’s current form.

Among the index cases, only 49% reported HRC. This proportion might signal underreporting, but it should be taken into account that it is impacted by the index cases in collectivities (e.g. school, kindergarten, nursing home) and companies for which local contact tracing is carried out for their contacts within that collectivity, which are not reported to the central contact center. Among the index cases that reported high-risk contacts, the mean number of HRC was around 2.7 and this number remained stable between January and September 2021. This appears to be low, especially taking into account the lifting of most nonpharmaceutical interventions impacting social contacts since June 2021 [40]. On the other hand, several measures that could reduce the number of HRCs remained mandatory, e.g. mask-wearing in public transport and shops. The number of identified contacts in other countries varies widely with only 1.15 in the US, 1.4 in the UK, and more than 17 in Taiwan [37, 41, 42]. Differences in nonpharmaceutical interventions, the definition of an HRC, the inclusion of low-risk contacts in the tracing system, coverage of the system, and compliance of the population all impact these numbers. It is essential that index cases report all their contacts to maximize the effect of contact tracing activities. Sensitization of the general population would be needed to improve the awareness about the importance of reporting all contacts and to find the willingness and trust to do so.

Our results support that contact tracing is a useful targeted public health tool that effectively results in finding cases and presumably in an earlier phase than compared to a system that would only rely on symptom-based testing and case finding. Overall the proportion of COVID-19 cases that were previously identified as HRC was steady around 24%. This proportion may be an underestimation, partly due to some HRC not reporting their NRN (14%) as well as due to contact tracing in companies and collectivities not included in the central tracing system. The latter may also have contributed to a possible overestimation of household transmission. Transmission at home was most often reported as the suspected place of infection by the index cases, 2 out of 3 reported HRC where household members and the SAR among household HRC was with 34% double as high as among non-household HRC.

Testing and quarantine of HRC are crucial measures to reduce the risk of transmission and to identify newly exposed HRC whenever an HRC becomes an index case. Overall 89% of the HRC got tested at least once. We reported a high overall SAR of 27%, which is partly thanks to the double testing strategy of the HRC, as many cases were only identified during their second test. On the other hand, not all HRC were reported, leading to an overrepresentation of household members, who have a higher positivity rate. Nevertheless, even with a SAR of 16% among non-household HRC, our results suggest that contact tracing is an effective way of case finding as the reported HRC (with NRN) represented only 6% of all tests in Belgium but 24% of all new cases.

Data of the adult population between January and September 2021 also illustrates that vaccinated and unvaccinated people complied with the testing strategy as respectively 92% vs 85% were tested at least once. All HRC received an officially recognized quarantine certificate for the quarantine period, with information about the mandatory and recommended measures but the follow-up of these measures is more difficult to evaluate.

In Belgium, the first vaccines were delivered on 28 December 2020. The campaign continued, in different phases. By the end of September 2021, 73% of the general population was fully vaccinated [43]. In contrast, only 36% of index cases were fully vaccinated at that time. The over-representation of unvaccinated persons among index cases is in line with the previously described protection of vaccination against infection among the Belgium population [14]. The proportion of HRC fully vaccinated was also lower than the proportion of fully vaccinated persons in the general population. This difference may be explained by the fact that the largest proportion of the unvaccinated population by the end of the study period were children and adolescents, who in general had a higher incidence when vaccination increased in the general adult population, causing a higher number of high-risk contacts in unvaccinated, compared to the oldest age groups who have the highest vaccination coverages. Furthermore, there might be clustering due to household members or friends who may tend to have a similar vaccination status. SAR among unvaccinated HRC are two times higher compared to vaccinated HRC, illustrating the protection of vaccination against infection during the first 9 months of 2021.

## Conclusion

Between January and September 2021, a robust system of contact tracing was in place in Belgium. The process of contact tracing, from a positive test of the index case to contact its HRC was mostly completed in a day. More than 90% of index cases and their HRC were contacted. Reduction of the delay between onset of illness and consultation can potentially further improve the effectiveness of contact tracing. The high SAR and the high proportion of cases previously registered as HRC are indications of effective early case detection through contact tracing. This remained the case after the successful roll-out of the vaccination campaign in 2021. Contact-tracing can be further improved by reducing the delay between onset of illness and medical consultation and an exhaustive reporting of HRC by all COVID-19 cases. Frequent and clear communication towards the general population about the importance of rapid testing and contact tracing and measures to keep the threshold for testing as low as possible are essential in this objective. Continued and enhanced data collection and analysis of contact tracing results can provide essential insight into this resource-intensive but important public health tool.

## Supplementary Information

**Additional file 1: Table S1.** Overview of the main changes in SARS-CoV-2 testing strategy in Belgium, January – September 2021. **Table S2.** Characteristics of COVID-19 index cases and high-risk contacts, Belgium, January – September 2021.

## Data Availability

The datasets supporting the conclusions of this article are available included within the article’s additional files.

## Abbreviations

COVID-19: Coronavirus disease 2019
HRC: High-risk contact
CC: Contact center
