# Supplementary material for: COVID-19 contact tracing in Belgium: main indicators and performance, January – September 2021
Source: Arch Public Health. 2022 Apr 13;80:118. doi: 10.1186/s13690-022-00875-6 (PMC9005619; doi:10.1186/s13690-022-00875-6)
Supplement: Supplementary file 1 — Additional file 1: Table S1. Overview of the main changes in SARS-CoV-2 testing strategy in Belgium, January – September 2021. Table S2. Characteristics of COVID-19 index cases and high-risk contacts, Belgium, January – September 2021. [file 13690_2022_875_MOESM1_ESM.docx]

**Supplementary files**

Supplementary Table 1: Overview of the main changes in SARS-CoV-2 testing strategy in Belgium, January – September 2021

| **Timing** | **Strategy** |
| --- | --- |
| Since May 2020 | Testing of all symptomatic patients that fulfilled the case definition of a possible case (previously only severe cases were tested) |
| 01 January 2021 | Two tests for travelers returning from high-risk areas abroad: as soon as possible after arrival and on day 7 after the day of return. |
| 25 January 2021 | Two tests for HRC: at the moment of identification and day 7 after the last high-risk contact. |
| 06 April 2021 | No retesting or quarantine of individuals within **90 days** of an initial positive test(except if serious symptoms require hospitalization). |
| 24 June 2021 | Fully vaccinated HRC are tested as soon as possible after identification. In case of a negative result, they are exempted from a second test and quarantine. |
| 01 July 2021 | Increased testing in the framework of the Digital Covid Certificate (giving access to international travel and large events): two PCR tests are offered free of charge to persons without full vaccination |
| 01 July 2021 | Asymptomatic HRC with a confirmed COVID-19 infection in the past **180 days** are exempt from quarantine and testing. |
| 01 July 2021 | Testing strategy travelers depending on test, recovery, vaccination certificate, and country of travel [44] |
| 12 July 2021 | Antigenic testing in the framework of Digital Covid Certificate additionally performed in pharmacies |
| 31 August 2021 | Fully vaccinated HRC who are tested negative immediately after a high-risk exposure receive a second PCR-test 7 days after last exposure (no quarantine between tests). |

Supplementary Table 2: Characteristics of COVID-19 index cases and high-risk contacts, Belgium, January – September 2021

|  | Index case reported to contact center No. (%) | Contacted index case No. (%) | High-risk contacts with NRN No. (%) |
| --- | --- | --- | --- |
| Age group: |  |  |  |
| 0-9 | 45.290(8.2) | 42.977(8.5) | 74.442(13) |
| 9-19 | 96.418(17.4) | 89.451(17.7) | 107.581(18.8) |
| 20-29 | 92.620(16.7) | 83.529(16.5) | 85.744(15) |
| 30-39 | 88.052(15.9) | 80.612(15.9) | 80.125(14) |
| 40-49 | 81.444(14.7) | 75.443(14.9) | 88.655(15.5) |
| 50-59 | 65.943(11.9) | 61.153(12.1) | 69.028(12) |
| 60-69 | 37.178(6.7) | 33.998(6.7) | 37.839(6.6) |
| 70-79 | 20.880(3.8) | 18.365(3.6) | 16.664(2.9) |
| 80+ | 19.856(3.6) | 15.876(3.1) | 6.406(1.1) |
| unknown | 7.500(1.4) | 5.015(1) | 6.934(1.2) |
| Sex: |  |  |  |
| M | 286.465(51.6) | 262.435(51.8) | 292.653(51) |
| F | 266.499(48) | 243.563(48.1) | 279.184(48.7) |
| U | 2.217(0.4) | 421(0.1) | 1.581(0.3) |
| Region of residence: |  |  |  |
| Brussels | 79.228(14.3) | 71.677(14.2) | 67.924(11.8) |
| Flanders | 293.267(52.8) | 271.318(53.6) | 338.406(59) |
| Wallonia | 178.634(32.2) | 161.869(32) | 165.047(28.8) |
| Unknown | 4.052(0.7) | 1.555(0.3) | 2.041(0.4) |
